# An Adjuvant-Based Approach Enables the Use of Dominant *HYG* and *KAN* Selectable Markers in Candida albicans

**DOI:** 10.1128/msphere.00347-22

**Published:** 2022-08-15

**Authors:** Sang On Park, Corey Frazer, Richard J. Bennett

**Affiliations:** a Molecular Microbiology and Immunology Department, Brown University, Providence, Rhode Island, USA; University of Georgia

**Keywords:** antibiotic selection, drug resistance, dominant marker, fungal pathogen, selectable marker

## Abstract

Candida albicans is a pathobiont fungus that can colonize multiple niches in the human body but is also a frequent cause of both mucosal and systemic disease. Despite its clinical importance, a paucity of dominant selectable markers has hindered the development of tools for genetic manipulation of the species. One factor limiting the utilization of dominant selectable markers is that C. albicans is inherently more resistant to antibiotics used for selection in other species. Here, we showed that the inclusion of suitable adjuvants can enable the use of two aminoglycoside antibiotics, hygromycin B and G418, for positive selection in C. albicans. Combining these antibiotics with an adjuvant, such as quinine or molybdate, substantially suppressed the background growth of C. albicans, thereby enabling transformants expressing *CaHygB* or *CaKan* markers to be readily identified. We verified that these adjuvants were not mutagenic to C. albicans and that *CaHygB* and *CaKan* markers were orthogonal to the existing marker *NAT1/SAT1*, and so provide complementary tools for the genetic manipulation of C. albicans strains. Our study also established that adjuvant-based approaches can enable the use of selectable markers that would otherwise be limited by high background growth from susceptible cells.

**IMPORTANCE** Only a single dominant selectable marker has been widely adopted for use in the opportunistic fungal pathogen Candida albicans. This is in stark contrast to model fungi where a repertoire of dominant markers is readily available. A limiting factor for C. albicans has been the high levels of background growth obtained with multiple antibiotics, thereby limiting their use for distinguishing cells that carry an antibiotic-resistance gene from those that do not. Here, we demonstrated that the inclusion of adjuvants can reduce background growth and enable the robust use of both *CaHygB* and *CaKan* markers for genetic selection in C. albicans.

## INTRODUCTION

Candida albicans is a common component of the human microbiome, often found inhabiting mucosal niches such as the gastrointestinal (GI) tract and the skin. However, this species is also an important cause of life-threatening systemic infections, and even with treatment with antifungal drugs mortality can reach 40% (1). Despite its importance as a human pathogen, genetic analysis of this species has lagged behind those of related yeast species such as Saccharomyces cerevisiae. Reasons for this include the fact that C. albicans isolates are naturally diploid (2), and while haploid forms have been identified these are not in common use for studies of commensalism or pathogenesis due to their decreased fitness (3). Moreover, C. albicans is a member of the CTG clade (or Ser1 subclade), where the CTG codon is translated as serine instead of leucine as in the universal genetic code (4, 5). This means that constructs often need to be codon optimized specifically for this species.

An additional limitation has been the paucity of dominant selectable markers available for C. albicans and related CTG clade species. Currently, only one such selectable system is extensively in use in C. albicans which involves the *SAT1/NAT1* gene for selection with the antibiotic nourseothricin (or clonNAT) (6–9). Recycling of the *SAT1* gene enables multiple C. albicans transformations with this marker (7), but this is time-consuming and chromosomal rearrangements can occur when site-specific recombination sequences are present in multiple positions in the genome (10). There is, therefore, a pressing need to identify additional dominant selectable markers that are complementary to that of *SAT1/NAT1*.

One major obstacle to the utilization of alternative selectable markers has been the fact that C. albicans often exhibits high levels of tolerance or resistance to antibiotics used for genetic selection in other yeast species. For example, the aminoglycoside antibiotics hygromycin B and kanamycin/G418 are frequently used for selection in S. cerevisiae using the selectable markers *HphMX* and *KanMX*, respectively (11), yet C. albicans exhibits high levels of background growth on both antibiotics. A codon-optimized hygromycin B gene (*CaHygB*) was previously developed for C. albicans (12) yet has not been widely adopted by the field due to background growth even in the presence of high levels of hygromycin B. Overexpression of an *IMH3* allele was also shown to provide resistance against mycophenolic acid in C. albicans but has not been further utilized due to issues with its implementation (13, 14).

Multiple studies have examined combinatorial mixtures of antifungal compounds to determine those that exhibit additive or synergistic inhibition of fungal growth. These include those of Vallieres et al. who recently showed that the combination of certain transport inhibitors with aminoglycoside antibiotics had a synergistic effect on inhibiting C. albicans growth (15, 16). Synergistic inhibition occurred when combining hygromycin B with inhibitors of amino acid transport (e.g., quinines) or sulfate transport (e.g., molybdate or chromate) (15, 16). These combinations were effective at blocking growth as transport inhibitors led to a decreased pool of amino acids that synergized with the ribosome-targeting aminoglycosides.

In this work, we examined whether adjuvants could enable the use of aminoglycoside antibiotics for a robust selection of dominant selectable markers in C. albicans. We showed that adjuvants can effectively work in tandem with hygromycin B and G418 to reduce the background growth of C. albicans. Moreover, the expression of *CaHygB* or *CaKan* genes was sufficient for the “clean” selection of C. albicans transformants on a medium containing the corresponding antibiotic supplemented with an adjuvant. *CaHygB* and *CaKan* are orthogonal for selection with *SAT1/NAT1* so that three dominant selectable systems are now available for C. albicans to facilitate genetic analysis of this important fungal pathogen. We also highlight that the use of appropriate adjuvants with different markers is expected to expand the utilization of antibiotics for other microbial species for which high background growth has limited the use of these antibiotics.

## RESULTS

### Analysis of combinatorial antifungal activities with hygromycin.

In this work, we examined whether the inclusion of adjuvants could potentiate the activity of antibiotics to which C. albicans has natural tolerance or resistance. Previous work showed that hygromycin B inhibits the growth of C. albicans cells (12), yet considerable background growth is observed when cells of the standard laboratory strain, SC5314, are grown on YPD medium at 30°C even with high levels of this drug (600 μg/mL). High levels of background growth limit the use of the *CaHygB* gene as a robust selectable marker in this species as transformations result in lawns of growth on hygromycin-containing medium (see below).

**FIG 1:**
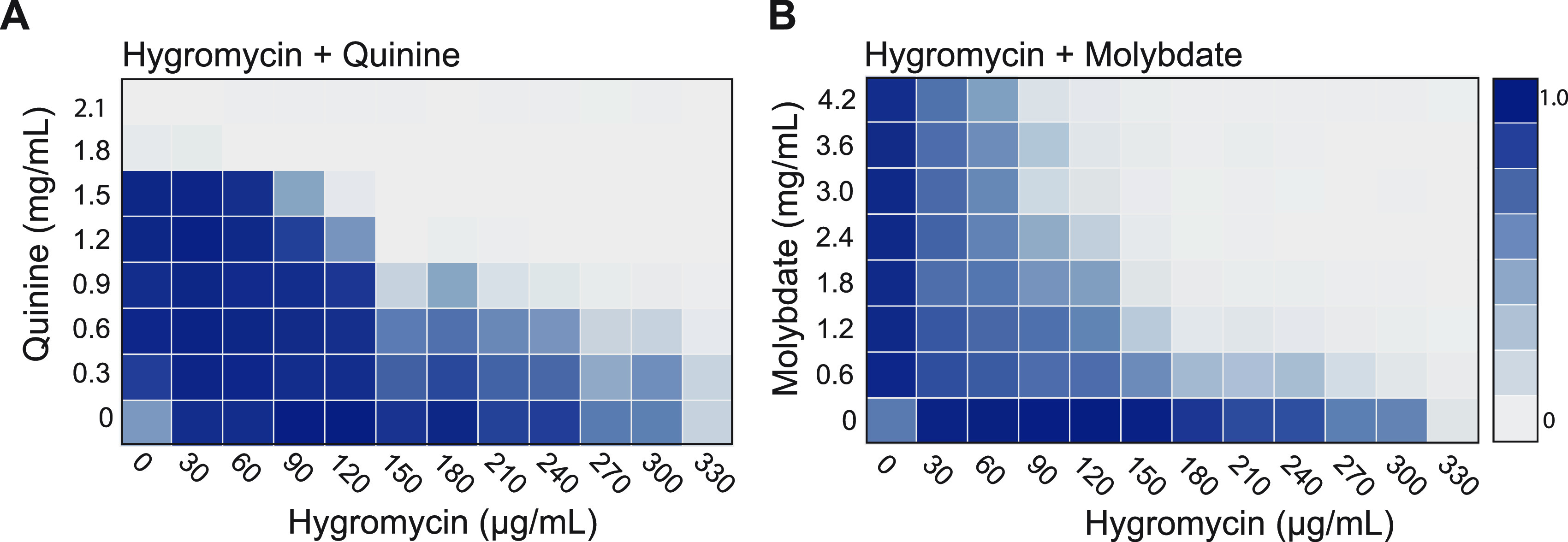
Quinine (A) and molybdate (B) act in tandem with hygromycin to inhibit C. albicans growth. 96-well checkerboard assays were performed in YPD containing the indicated drug concentrations. Plates were incubated at 30°C with orbital shaking, and OD_600_ was recorded for each well every 15 min for 24 h. The relative growth of each well was normalized to the growth of the well with the highest OD.

Vallieres et al. (15) recently reported that certain adjuvants can be used in combination with hygromycin B to reduce the growth of susceptible C. albicans cells. Hygromycin B is an aminoglycoside that targets ribosome function and quinine, molybdate, and chromate were identified as three compounds that showed additive or synergistic interactions with this antibiotic, potentially due to depletion of the amino acid pool (15). We carried out 96-well checkerboard assays to examine interactions between these chemicals using assays with SC5314 cells grown in YPD medium at 30°C and measured absorbance every 15 min for 24 h. Both quinine and molybdate acted in tandem with hygromycin to substantially inhibit the growth of C. albicans cells compared to hygromycin or adjuvant alone (Fig. 1A and B).

### Adjuvants enable the use of *CaHygB* as a dominant selectable marker with hygromycin.

**FIG 2:**
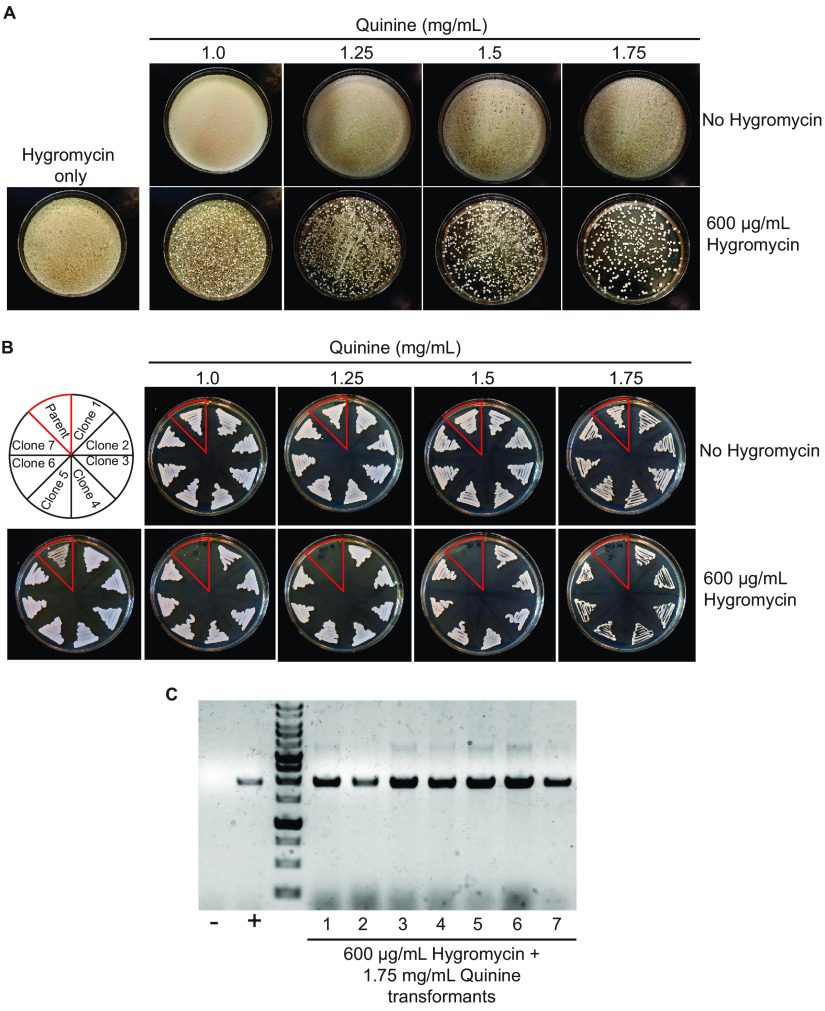
Quinine acts in combination with hygromycin for the selection of a *CaHygB* marker. (A) Transformation with a linearized construct carrying the *CaHygB* gene was carried out and plated on YPD containing different combinations of hygromycin and quinine as indicated. Plates were incubated at 30°C for 72 h. (B) The parental SC5314 C. albicans strain and seven colonies from the plate containing 0.6 mg/mL hygromycin + 1.75 mg/mL quinine were subcultured on plates containing different combinations of hygromycin and quinine as indicted. Plates were incubated at 30°C for 24 h. (C) PCR check for successful integration of the *CaHygB* gene into the C. albicans genome. All seven transformant colonies were positive, confirming the successful integration of *CaHygB*. The parental SC5314 strain was used as a negative control, and a strain carrying pSFS2A-SAT1 similarly integrated at the *MAL2* promoter was used as a positive control.

The *CaHygB* gene was previously codon-optimized for C. albicans and encodes a hygromycin B phosphotransferase activity that inactivates hygromycin (12). We integrated *CaHygB* into the vector pSFS2A, replacing the *SAT1* gene originally present in this vector (7). This plasmid was linearized within the *MAL2* promoter region for integration at the endogenous *MAL2* locus in C. albicans (see Materials and Methods). Transformations were carried out using a standard lithium acetate/polyethylene glycol protocol and transformation mixtures plated to YPD supplemented with hygromycin alone or hygromycin containing different amounts of quinine (0 to 1.75 mg/mL). As shown in Fig. 2A, transformation plates containing only hygromycin (600 μg/mL) or quinine (up to 1.75 mg/mL) generated lawns of cells where individual colonies were not visible. In contrast, individual colonies were evident on all the hygromycin plates supplemented with quinine, and background growth was essentially abolished on hygromycin plates containing 1.5 or 1.75 mg/mL quinine (Fig. 2A).

Colonies from the hygromycin + 1.75 mg/mL quinine plate were subcultured on hygromycin plates supplemented with different quinine concentrations, together with the parental SC5314 control. These plates showed that colonies from the transformation plate regrew on each of the test plates while SC5314 control cells grew on hygromycin alone but not on any of the hygromycin + quinine plates (Fig. 2B). Analysis of the transformation colonies by PCR confirmed that all the test colonies from hygromycin+quinine plates contained the construct correctly integrated at the *MAL2* locus (Fig. 2C).

These results demonstrated that hygromycin alone is limited in its use for the direct selection of C. albicans transformants expressing the *CaHygB* marker. However, the combination of hygromycin with a suitable adjuvant, such as quinine, enables the robust selection of transformant colonies with little or no background growth.

### Analysis of combinatorial antifungal activities with G418.

Given that adjuvants can decrease the growth of susceptible C. albicans cells in a hygromycin-containing medium, we examined whether adjuvants could similarly enable selection with a second aminoglycoside antibiotic, G418. Many fungi are susceptible to G418 (Geneticin), which is closely related to kanamycin A, and the *KanMX* module has been extensively used as a selectable marker for this antibiotic in S. cerevisiae (11, 17). The *KanMX* module utilizes the *kan^R^* gene from the E. coli transposon Tn903 to provide resistance against G418 (11).

**FIG 3:**
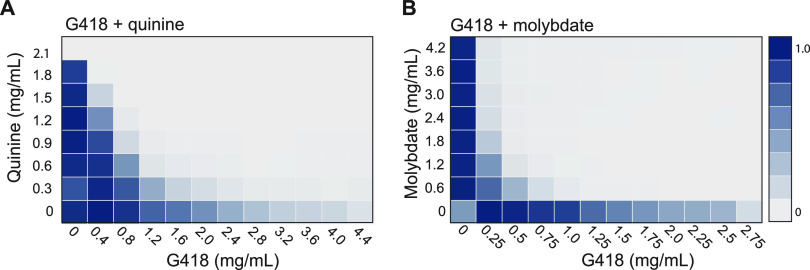
Quinine (A) and molybdate (B) act in tandem with G418 to inhibit C. albicans growth. The 96-well checkerboard assays were performed in YPD containing the indicated drug concentrations. Plates were incubated at 30°C with orbital shaking, and OD_600_ was recorded for each well every 15 min for 24 h. The relative growth of each well was normalized to the growth of the well with the highest optical density (OD).

To determine whether adjuvants that synergize with hygromycin also work with G418, checkerboard assays were performed with SC5314 cells grown in the presence of G418 together with various concentrations of quinine or molybdate. Strikingly, both compounds worked as adjuvants in combination with G418 (Fig. 3A and B) and revealed that quinine is effective when used with two distinct aminoglycosides.

### Adjuvants enable the use of *CaKan* as a dominant selectable marker for G418.

**FIG 4:**
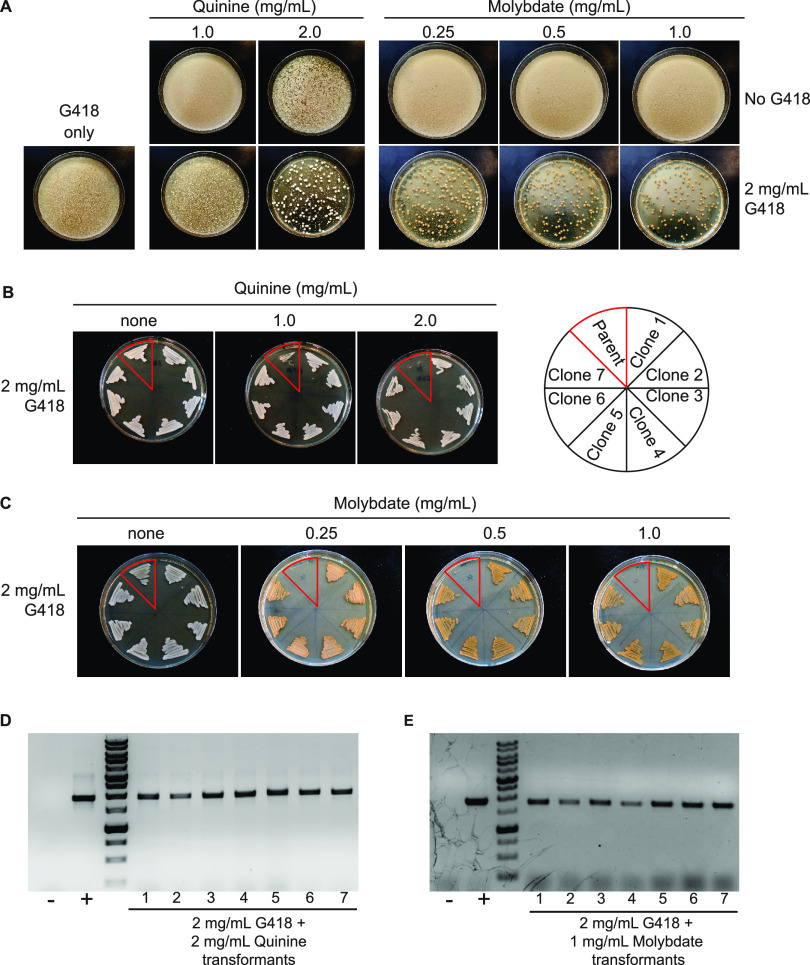
Quinine and molybdate act in combination with G418 for the selection of a *CaKan* marker. (A) Transformation of a plasmid carrying the *CaKan* gene was performed and transformants were cultured on YPD medium containing different combinations of G418, quinine, and molybdate as indicated. Plates were incubated at 30°C for 72 h. (B) The parental SC5314 strain and seven transformant colonies (taken from the plate containing 2 mg/mL G418 + 2 mg/mL quinine) were cultured on plates containing G418 and quinine as indicated. Plates were incubated at 30°C for 24 h. (C) In a control SC5314 strain, seven transformants from G418/molybdate selection were subcultured on plates containing different G418 and molybdate concentrations as indicated. Plates were incubated at 30°C for 24 h. (D) PCR analysis of seven transformants from G418/quinine and seven colonies from G418/molybdate were tested and all 14 contained the *CaKan* cassette correctly integrated at the *MAL2* locus. The parental SC5314 strain was used as a negative control and a strain containing a pSFS2A-SAT1 construct similarly integrated at the *MAL2* promoter was used as a positive control.

We are not aware of any reports of G418 having been used for antibiotic selection in C. albicans. To determine if the *Kan^R^* gene from the Tn903 transposon is functional in C. albicans, we codon optimized this gene for this species and used the resulting *CaKan* gene to replace the *SAT1* marker in pSFS2A (see Materials and Methods). This construct was again targeted for integration at the *MAL2* locus by linearization within the *pMAL2* promoter and transformants grown on YPD plates supplemented with different combinations of G418 with either quinine or molybdate as an adjuvant. As shown in Fig. 4A, the inclusion of either adjuvant was found to result in a substantial reduction in background growth on transformation plates containing G418 compared to the G418 antibiotic alone. Thus, G418 + 2 mg/mL quinine or G418 + 0.5 to 1 mg/mL molybdate gave very low background growth on which transformation colonies were evident (Fig. 4A). We note that colonies grown on a molybdate-containing medium were a darker color, but these colonies form the typical off-white color when restruck on media lacking molybdate.

Colonies from G418+quinine or G418+molybdate transformation plates were subcultured onto G418-containing plates with or without these adjuvants. In each case, colonies from transformations grew on G418+adjuvant plates whereas SC5314 control cells did not grow on plates containing both antibiotic and adjuvant (Fig. 4B). To confirm that colonies from G418+adjuvant transformations contained the pSFS2A-*CaKan* construct, 7 random colonies were analyzed from both G418+quinine and G418+molybdate plates. PCR analysis of these colonies confirmed that each transformant carried the *CaKan* construct correctly integrated at the *MAL2* locus, demonstrating that this selection method can be successfully used for genetic studies in C. albicans.

### *CaHygB* and *CaKan* are orthogonal markers to *SAT1/NAT1* in C. albicans.

**FIG 5:**
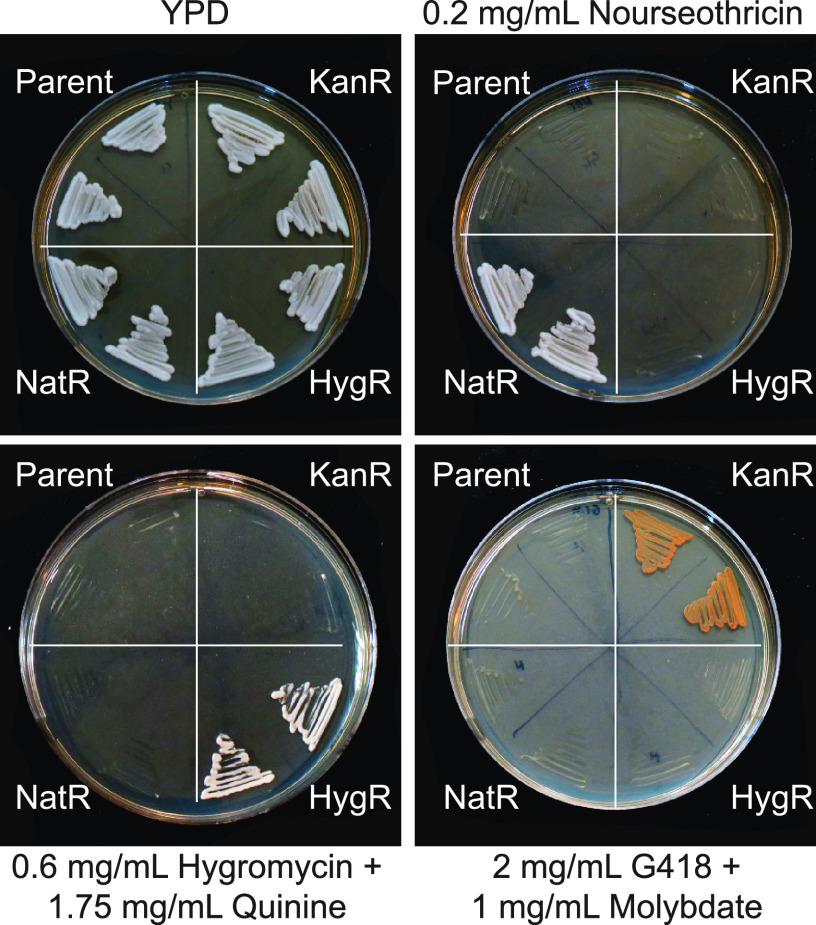
*SAT1*, *CaHygB*, and *CaKan* are three orthogonal selection markers in C. albicans. C. albicans strains carrying the *SAT1* nourseothricin marker, the *CaHygB* hygromycin marker, the *CaKan* G418 marker, or no selection marker were grown on YPD, YPD + 0.2 mg/mL NAT, YPD + 0.6 mg/mL hygromycin (+1.75 mg/mL quinine), or YPD + 2 mg/mL G418 (+2 mg/mL quinine). Only cells carrying the corresponding selection marker grew on the appropriate antibiotic. Plates were incubated at 30°C for 24 h.

To establish that *CaHygB* and *CaKan* can be used as complementary markers to *SAT1/NAT1* in C. albicans, we analyzed the growth of SC5314 cells carrying each of these markers (as well as control SC5314 cells) on YPD, YPD+nourseothricin, YPD+hygromycin (+adjuvant) and YPD+G418 (+adjuvant). As shown in Fig. 5, only cells carrying a dominant selectable marker were able to grow on the corresponding antibiotic-containing medium. Thus, *CaHygB*, *CaKan*, and *SAT1/NAT1* represent three orthogonal markers that can be used for genetic selection experiments in C. albicans, including the robust identification of transformants.

### Media containing adjuvants are not mutagenic to C. albicans.

**FIG 6:**
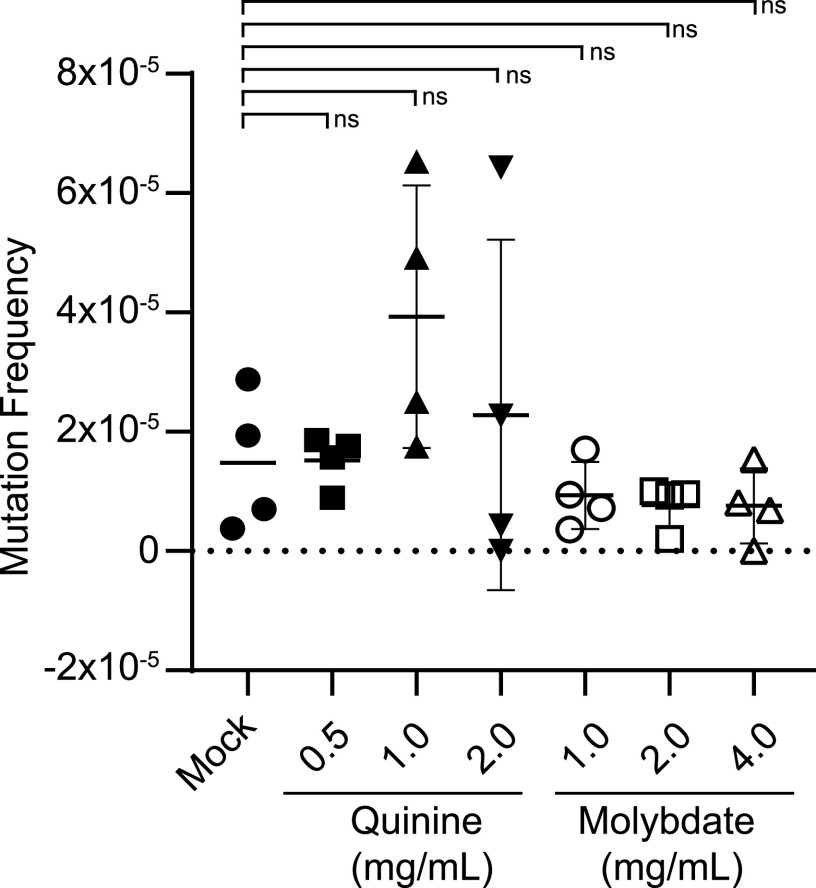
Quinine and molybdate are not significantly mutagenic to C. albicans. A C. albicans strain heterozygous for *URA3* was incubated on plates containing different concentrations of adjuvants as indicated. Colonies were resuspended after 72 h of incubation at 30°C and plated to 5-FOA and YPD plates. Mutagenicity was calculated by dividing the number of colonies on 5-FOA plates by the number of colonies on YPD plates multiplied by 10,000. Each condition includes 4 replicates. The significance test was performed with Welch’s *t* test with a significance value of 0.05.

To examine if adjuvants are mutagenic to C. albicans, we used a counterselection assay using a strain heterozygous for *URA3*. Loss of *URA3* enables C. albicans growth on a medium containing 5-fluoroorotic acid (5-FOA) and can therefore be used to monitor levels of mutagenesis. SC5314 cells were grown on plates containing various concentrations of adjuvants at 30°C for 72 h and replated on SCD plates supplemented with 5-FOA or YPD plates. There was no significant difference in the proportion of cells that were 5-FOA resistant between control and adjuvant-containing conditions (Fig. 6). These results establish that quinine and molybdate are not highly mutagenic toward C. albicans and can therefore be utilized for genetic selection assays.

## DISCUSSION

Mechanistic studies of C. albicans biology have lagged those of model yeast such as S. cerevisiae, in part due to a more limited set of genetic tools. While new experimental approaches continue to expand the toolset for C. albicans, including CRISPR-based methods for more facile engineering of the species (18–22), there remain several technical limitations compared to model organisms. This includes the fact that only a single dominant selection system, *SAT1/NAT1* for growth on nourseothricin, has been widely adopted by the field, whereas multiple such markers exist for other fungal species with 17 such markers reported for S. cerevisiae (23). The presence of only one dominant marker for C. albicans is particularly limiting given that isolates are diploid, so this marker often must be recycled for gene deletions. This is particularly true in clinical isolates for which auxotrophic derivatives are not readily available.

Here, we reported that *CaHygB* or *CaKan* markers can be robustly used as genetic markers in C. albicans when cells are grown on the appropriate antibiotic (hygromycin B or G418, respectively) and when suitable adjuvants are present to reduce the level of background growth. *CaHygB* was previously reported as a dominant marker for C. albicans in 2010 (12) yet has not been widely adopted by the field which reflects the high levels of background growth observed even with high levels of hygromycin (see Fig. 2). A gene providing resistance to G418/kanamycin has not been previously reported as a selectable marker for C. albicans, yet the *KanMX* gene has been extensively used with this antibiotic in S. cerevisiae (11, 23, 24). We demonstrated that *CaKan*, a codon-optimized version of the bacterial Tn 903 phosphotransferase (11), can be utilized in C. albicans by culturing cells on G418 together with appropriate adjuvants. Transformants carrying *CaHygB* or *CaKan* can, therefore, be selected using this combinatorial approach, and both *CaHygB* and *CaKan* are orthogonal to *SAT1/NAT1* such that three independent antibiotic markers are now available for this important species.

Both hygromycin and G418 are aminoglycoside antibiotics that inhibit translation by targeting the ribosome, and previous studies indicated that hygromycin activity against C. albicans is synergistic when combined with transport inhibitors (15, 16). The two adjuvants utilized in the current work were molybdate, a sulfate transport inhibitor (16), and quinine, an amino acid transport inhibitor (25). These adjuvants showed combinatorial inhibition of C. albicans with hygromycin and G418 antibiotics in our assays. Certain transport inhibitors can synergize with aminoglycosides due to an increased rate of translational errors (16). Several other transport inhibitors, such as chromate, have also been shown to synergize with aminoglycosides suggesting they could be used as alternatives to quinine/molybdate for enabling antibiotic selection (15, 16). However, we note that both quinine and molybdate are inexpensive and did not cause an increased mutation frequency in C. albicans, making them suitable for adoption for genetic selection assays.

In addition to the development of key reagents for C. albicans research, our study suggests that adjuvant treatment is a powerful approach for enabling the use of antibiotic markers for other microbial (or even nonmicrobial) species. We highlighted that the major limitation with using aminoglycosides, such as hygromycin and G418 in C. albicans, was not the lack of a functional antibiotic resistance gene for selection but the high levels of background growth. This was particularly true when using high cell densities during transformations. Thus, adjuvants that promote antibiotic efficacy ensure that these antibiotics can be used with the corresponding selectable markers. Future studies will look to determine if other dominant selectable markers can be established for use in C. albicans or other CTG clade species, and to more boradly establish combinatorial agents for robust selection of genetically marked strains. Moreover, we are interested to see if the adjuvant approach described here will be applied to other systems for which improved antibiotic selectivity is desired.

## MATERIALS AND METHODS

### Reagents and media.

The standard C. albicans laboratory strain SC5314 was used throughout this study. All strains were grown at 30°C in YPD medium (10 g/L yeast extract [Gibco], 10 g/L peptone [Gibco], and 20 g/L glucose [Fisher Scientific]). Hygromycin B (GoldBio H-270-5), G418 (GoldBio G-418-5), quinine hydrochloride dihydrate (Acros Organics AC163720250), and sodium molybdate (VI) dihydrate (Sigma-Aldrich 331058) were dissolved in water and added at the concentrations indicated in the text. Nourseothricin (Jena BioScience) was used at 200 μg/mL.

### Plasmid construction.

**pSFS2A-CaHygB.** The *CaHygB* gene together with the *TEF2* promoter and *ACT1* terminator was PCR amplified from pYM70 (12) using oligonucleotides 1653 and 1651 (Table 1). A 716 bp fragment of pSFS2A extending from upstream of the HindIII site in the FLP recombinase gene through its *ACT1* terminator was PCR amplified using oligonucleotides 1669 and 1652. The two fragments were mixed, and PCR performed with oligonucleotides 1669 and 1651 to generate the corresponding fusion product. This fusion product was then digested with HindIII and PstI and cloned into pSFS2A digested with these enzymes to generate pRB195. To remove an XhoI restriction site in the *TEF2* promoter of pRB195 the plasmid was digested with XhoI, the single-stranded overhangs filled with the Klenow fragment of T7 DNA polymerase (NEB) and ligated with DNA ligase (NEB) to create pRB196. To remove a SacI site in the *CaHygB* gene, a silent T285G substitution was made using the mutagenic oligonucleotides MutF and MutR to generate pRB197.

**TABLE 1:** Oligonucleotides used in this study

Oligo #	Oligonucleotide name	Sequence^*a*^
1653	pYM70 (5180)	CCGCTGCTAGGCGCGCCGTGCCACCTGACGTCGTATAGTG
1651	pYM70 (7290) PstI	GGCGCCCTGCAGCATGATAATAATGGTTTCTTAGACGTC
1669	pSFS2A (2100) for	CTTAGTCAGATCGTACAATAAAGC
1652	pSFS2A (2800) rev	CACGGCGCGCCTAGCAGCGGATAGAAATGCCTTGGGTGGC
7128	pSFS2A for CaKan fwd GGA-BsaI	GGACCGGGTCTCTTCATATGGGAAAAGAAAAGACCCAC
7129	pSFS2A for CaKan rev GGA-BsaI	GGACCGGGTCTCTTATGTCAGAAGAACTCATCTAACATCAAATGG
7126	CaKan to pSFS2A fwd GGA-BsaI	GGACCGGGTCTCTCATATGTGAAGTGTGAAGGGG
7127	CaKan to pSFS2A rev GGA-BsaI	GGACCGGGTCTCTATGAGAACCGTTATCGATAACTAAAG
3727	Mal2 integration check fwd	GTCGTTGGGGTTGATTTGTTTC
3811	Mal2 integration check rev	GTCTTCCAATGTGAGATTTTGGGCC
MutF	SacI mut Fwd	CTTATTGTATTTCAAGAAGAGCGCAAGGTGTTACTTTGCAAG
MutR	SacI mut Rev	CTTGCAAAGTAACACCTTGCGCTCTTCTTGAAATACAATAAG

a Blue, restriction enzyme cut site; green, overlap for PCR fusion; red, substitution to remove SacI site; underlined, Golden Gate Assembly overlaps.

**pSFS2A-CaKan.** Oligonucleotides 7128 and 7129 were used to PCR amplify pSFS2A except for the *SAT1* ORF and to incorporate BsaI restriction sites on each end. A *Candida-*optimized KanR gene was synthesized (Twist BioScience; pRB1799) and PCR amplified using oligonucleotides 7126/7127, which incorporated BsaI sites to generate complementary overlaps to those on the plasmid amplicon. These two fragments were digested and ligated using the standard NEB Golden Gate Assembly protocol.

The nucleotide sequences of pSFS2A-HygR and pSFS2A-CaKan were confirmed by next-generation sequencing (Plasmidsaurus).

**C. albicans transformations.** Yeast transformations were performed using a lithium acetate/polyethylene glycol method as described (26). Plasmids pSFS2A, pSFS2A-CaHygB, and pSFS2A-CaKan were linearized with BsrGI to target constructs for integration at the C. albicans *MAL2* promoter. Integration at the *MAL2* promoter was confirmed by colony PCR using oligonucleotides 3727 and 3811.

### Checkerboard assays.

In each well of a 96-well plate, 100 μL of sterile water containing 2× the indicated drug concentrations were mixed with C. albicans cells (0.02 optical density at 600 nm [OD_600_] units; 10^5^ cells) in 100 μL of 2× YPD medium. Plates were incubated in a BioTek Epoch2 microplate spectrophotometer at 30°C for 24 h with orbital shaking and absorbance measured at OD_600_.

### Mutagenicity assays.

A C. albicans SC5314 strain heterozygous for *URA3* (RBY1177 (27)) was utilized for mutagenicity assays. 100 μL of cell suspension (5 × 10^−5^ OD_600_ units) was cultured on YPD plates supplemented with various concentrations of adjuvants at 30°C for 72 h. On YPD plates supplemented with 2 mg/mL quinine, 100 μL of 5 × 10^−4^ (or 5 × 10^−3^) OD_600_ units of cell suspension was plated and incubated at 30°C for 72 h due to delayed growth. Colonies were picked into cell suspension, then diluted to an OD_600_ of 0.5, and 100 μL of cell suspension was plated on SCD plates supplemented with 0.8 mg/mL of 5-fluoroorotic acid (5-FOA; Zymo Research). The same cell suspension was diluted to an OD_600_ of 5 × 10^−5^, and 100 μL of the solution was plated on YPD plates. Colonies were counted after 3 days of incubation at 30°C.

### Data availability.

The nucleotide sequences of pSFS2A-HygR and pSFS2A-CaKan, as well as the plasmids themselves, are available at Addgene (ID numbers 189564 and 189565, respectively).
